# Reconciling the Evidence on Serum Homocysteine and Ischaemic Heart Disease: A Meta-Analysis

**DOI:** 10.1371/journal.pone.0016473

**Published:** 2011-02-02

**Authors:** David S. Wald, Joan K. Morris, Nicholas J. Wald

**Affiliations:** Wolfson Institute of Preventive Medicine, Barts and the London School of Medicine, Queen Mary University of London, London, United Kingdom; Lerner Research Institute, Cleveland Clinic, United States of America

## Abstract

**Background:**

Results from genetic epidemiological studies suggest that raised serum homocysteine is a cause of ischaemic heart disease, but the results of randomised trials suggest otherwise. We aimed to update meta-analyses on each type of study using the latest published data and test a hypothesis based on antiplatelet therapy use in the trials to explain the discrepancy.

**Methods and Findings:**

Meta-analyses of ischaemic heart disease using (i) 75 studies in which the prevalence of a mutation (C
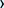
T) in the MTHFR gene (which increases homocysteine) was determined in cases (22,068) and controls (23,618), and (*ii*) 14 randomised trials (39,597 participants) of homocysteine lowering and ischaemic heart disease events. The summary estimates from the two analyses were compared. Meta-analysis of the MTHFR studies showed a statistically significantly increased risk of ischaemic heart disease in TT compared with CC homozygotes; odds ratio 1.16 (1.04 to 1.29) for a 1.9 µmol/L homocysteine difference (TT minus CC). Meta-analysis of randomised trials showed no significant reduction in IHD risk from folic acid; relative risk 1.00 (0.93 to 1.08), despite a reduction in homocysteine of 3.3 µmol/L. There was a statistically significant difference in risk reduction between the 5 trials with the lowest prevalence of antiplatelet therapy (60% on average, usually aspirin), RR 0.93 (0.84 to 1.05) and the 5 trials with the highest prevalence (91% on average), RR 1.09 (1.00 to 1.19), p = 0.037 for the difference.

**Conclusion:**

Discordant results from MTHFR studies and randomised trials could be explained by aspirin reducing or negating the anti-platelet effect of lowering homocysteine. On this basis, folic acid would have a role in the primary prevention of ischaemic heart disease, when aspirin is not taken routinely, but not in secondary prevention, when it is routine.

## Introduction

There is uncertainty over whether raised serum homocysteine concentrations cause ischaemic heart disease. Two types of study provide evidence: (i) case control studies of the prevalence of the methylenetetrahydrofolate reductase (MTHFR) gene polymorphism (a common genetic variant that leads to moderate increases in serum homocysteine levels) among people with and without ischaemic heart disease [1], [2] and (ii) randomised trials of B vitamins, which lower serum homocysteine, in the prevention of ischaemic heart disease events.[3] The randomised trials should be a valid test of the hypothesis that homocysteine causes ischaemic heart disease, provided risk can be reversed within a few years – the duration of most of the trials.

The MTHFR studies, themselves, provide evidence of causality comparable to that obtained from the randomised controlled trials, in that the serum homocysteine differences in people with and without the polymorphism occur as a result of a randomly allocated genetic mutation and the two groups would not be expected to differ in other respects. While it is theoretically possible that B vitamins influence ischaemic heart disease risk through means other than homocysteine, none have been shown. Meta-analyses of MTHFR studies show a statistically significantly higher risk of ischaemic heart disease in TT homozygotes (the variant with higher homocysteine)[2]–[4] than in CC homozygotes (the variant with lower homocysteine), but meta-analyses of randomised trials of homocysteine lowering have indicated that folic acid, which lowers homocysteine does not reduce the risk of ischaemic heart disease.[3], [5]

Additional MTHFR studies and randomised trials have been published since the last meta-analyses. [3]–[5] We here update the meta-analyses to provide a quantitative comparison between the two types of study and investigate a possible explanation (based on concomitant use of antiplatelet therapy in the trials) that could explain the discrepant results.

## Methods

We updated our previous meta-analyses [2] of (i) MTHFR studies that reported the prevalence of the TT, CT and CC genotypes in ischaemic heart disease cases and controls and (ii) MTHFR studies that reported serum or plasma homocysteine level according to genotype in individuals without a history of cardiovascular disease. We adopted our previous search strategy [2] but included only studies on ischaemic heart disease and extended the inclusion of studies to those published up to July 2010. The search identified an additional 64 MTHFR studies (114 in total) since the previous meta-analyses (Figure S1). We updated our previous meta-analysis of randomised placebo-controlled trials of serum homocysteine reduction and ischaemic heart disease events [3] including trials published up to July 2010. The search identified an additional 7 trials (14 in total) since the previous meta-analysis. Data were extracted independently by two investigators and the data-sets cross-checked.

In the MTHFR studies patients with ischaemic heart disease had myocardial infarction or angiographically confirmed coronary artery occlusion (>50% of the luminal diameter). The control groups were from the general population or patients who underwent coronary angiography but had normal coronary arteries. Patients with mild angiographic coronary artery disease (<50% occlusion of the luminal diameter) who were, in some studies, classified as control subjects were excluded from the meta-analysis. We restricted our analysis to studies in which MTHFR polymorphisms (TT, CT or CC) were confirmed by DNA testing. The studies therefore compared the risk of ischaemic heart disease in people with higher (TT) and lower (CC) homocysteine levels, the two groups determined at random through natural allele assortment.

In each MTHFR study we determined the odds ratio for ischaemic heart disease in TT versus CC homozygotes, and separately in CT versus CC, in cases and controls. We used a random effects model to derive summary odds ratios from combinations of studies to take account of heterogeneity across studies. We performed a meta-regression analysis of odds ratio (TT versus CC) against the homocysteine difference between TT and CC in studies where both were reported. From studies that reported serum homocysteine according to genotype in individuals without a history of cardiovascular disease, we calculated summary mean serum homocysteine differences (TT minus CC and CT minus CC), weighting studies by the inverse of the variance.

In the randomised trials we calculated the relative risk of ischaemic heart disease (death or non-fatal myocardial infarction) in each trial and used a random effects model to derive a summary relative risk estimate from combinations of trials. A pre-specified analysis was performed on the results of the trials in which the use of antiplatelet therapy was reported, separating them in to the half with the lowest and the half with the highest prevalence of antiplatelet therapy. STATA software (version 10) was used for all analyses.

## Results

### Studies of MTHFR mutation

**Figure 1:**
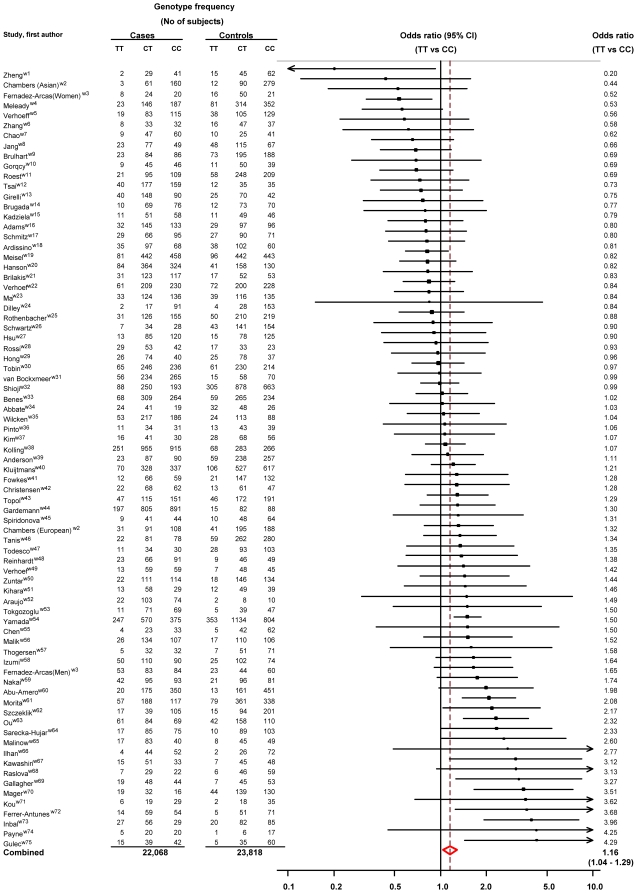
Meta-analysis of MTHFR studies and ischaemic heart disease: odds ratios (95% confidence intervals) for TT versus CC homozygotes.

The database search identified 75 MTHFR studies comparing the prevalence of TT with CC homozygotes in cases with ischaemic heart disease (22, 068 in total) and controls (23,618). Figure 1 shows the odds ratios for TT versus CC, with the studies ranked in order of increasing effect. The summary odds ratio was 1.16 (95% confidence interval 1.04 to 1.29; p = 0.006), so risk was 16% higher in TT than CC. The summary estimate for CT versus CC was 1.04 (0.99 to 1.10). There was significant heterogeneity between studies (I^2^ = 49.9%, p<0.001). Figure S2 is a plot of the standard error of the log odds ratio (TT versus CC) against the odds ratio for each MTHFR study (a funnel plot). The symmetry of the plot provides evidence against publication bias. Citations of the relevant published articles are given in Table S1.

**Figure 2:**
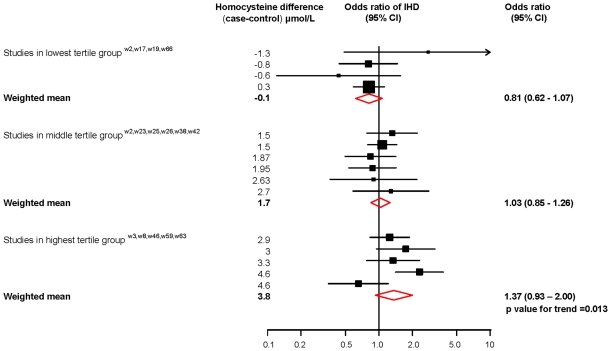
Dose-response relationship between odds ratio of ischaemic heart disease and difference in serum homocysteine concentrations between TT and CC homozygotes from meta-analysis of MTHFR studies.

Figure 2 shows a plot of the odds ratio of ischaemic heart disease against the serum homocysteine difference between TT and CC among control subjects in the 14 studies that reported these data. The plot ranks the studies by observed serum homocysteine difference and stratifies them into three groups according to serum homocysteine difference. The studies with similar homocysteine concentrations in the TT and CC groups showed no increased risk of ischaemic heart disease, whereas those with higher homocysteine in the TT group tended to have an increased risk. The odds ratio increased by 1.13 (1.00 to 1.27) for a 1 µmol/L increase in serum homocysteine (p = 0.013).

Table S2 lists 53 studies (36 167 participants), including the 14 from figure 2, which reported serum homocysteine but not risk of ischaemic heart disease according to MTHFR genotype in people without a history of cardiovascular disease. Citations of the relevant published articles are given in Table S3. The average serum homocysteine difference for TT minus CC was 1.9 µmol/L (1.5 to 2.2). Thus the expected dose-response relationship for TT versus CC is a relative risk of 1.16 for a 1.9 µmol/L serum homocysteine increase - lower than our previous published estimate of 2.7 µmol/L, [2] which included estimates from people with cardiovascular disease.

### Randomised Trials

The database search identified 14 randomised placebo-controlled trials of B vitamins on serum homocysteine reduction (Table 1), including 39,597 participants recording 3233 ischaemic heart disease events (cardiac death or non-fatal myocardial infarction). Citations for the relevant published trials are given in Table S4.

**Table 1:** Characteristics of randomised trials of serum homocysteine lowering on ischaemic heart disease events.

Study (country)*	Number of Participants	Previous Disease	Dose of B vitamin (mg)			Percentage Antiplatelet use (%)†	Follow up (months)
			**Folic acid**	**B12**	**B6**		
CHAOS - 2 (UK)^z1^	1882	IHD	5.0	-	-	NR	20
WAFACS (USA)^z2^	5442	CVD	2.5	1.0	50	51	88
VISP (USA)^z3^	3680	Stroke	2.5	0.4	25	NR	20
ASFAST (Australia)^z4^	315	Renal	15.0	-	-	22	43
Goes (Netherlands)^z5^	593	IHD	0.5	-	-	NR	42
Swiss Heart (Switzerland)^z6^	553	IHD	1.0	0.4	10	94	6
WENBIT (Norway)^z7^	3090	IHD	0.8	0.4	40	90	38
HOPE-2 (Canada)^z8^	5522	CVD	2.5	1.0	5	80	60
NORVIT (Norway)^z9^	3749	IHD	0.8	0.4	40	90	36
SEARCH (UK)^z10,z15^	12064	IHD	2.0	1.0	-	91	84
Lange* (Germany)^z11^	636	IHD	1.2	0.6	48	100	6
DIVINe (Canada)^z12^	238	Diabetic Renal	2.5	1.0	25	62	32
HOST (USA)^z13^	2056	Renal	40.0	2.0	100	40	38
Righetti* (Italy)^z14^	88	Renal	5.0	0.5	250	NR	29

*first author if study not named.

† aspirin and/or another antiplatelet drug.

IHD – ischaemic heart disease, CVD – Cardiovascular Disease.

NR – not reported.

**Figure 3:**
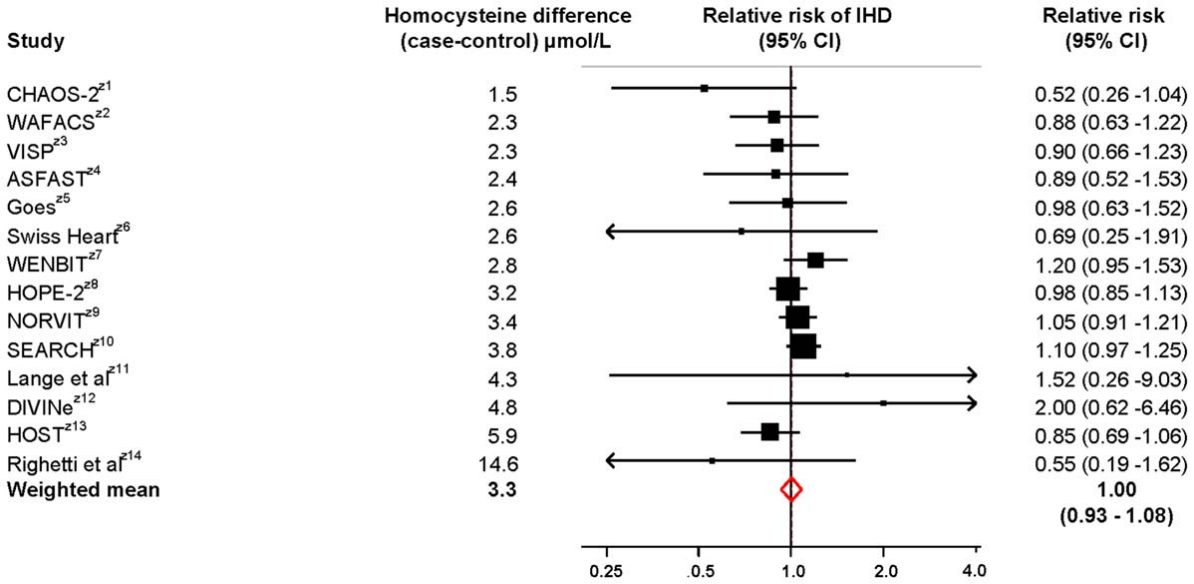
Meta-analysis of randomised trials of serum homocysteine reduction on ischaemic heart disease events (cardiac death and non-fatal myocardial infarction). * first author given if study not named.

Figure 3 shows a meta-analysis plot of the 14 trials. The summary relative risk was 1.00 (0.93 to 1.08) for a mean serum homocysteine reduction of 3.3 µmol/L. There was no significant heterogeneity between the trial results (I^2^ = 11.1%, p = 0.421).

**Figure 4:**
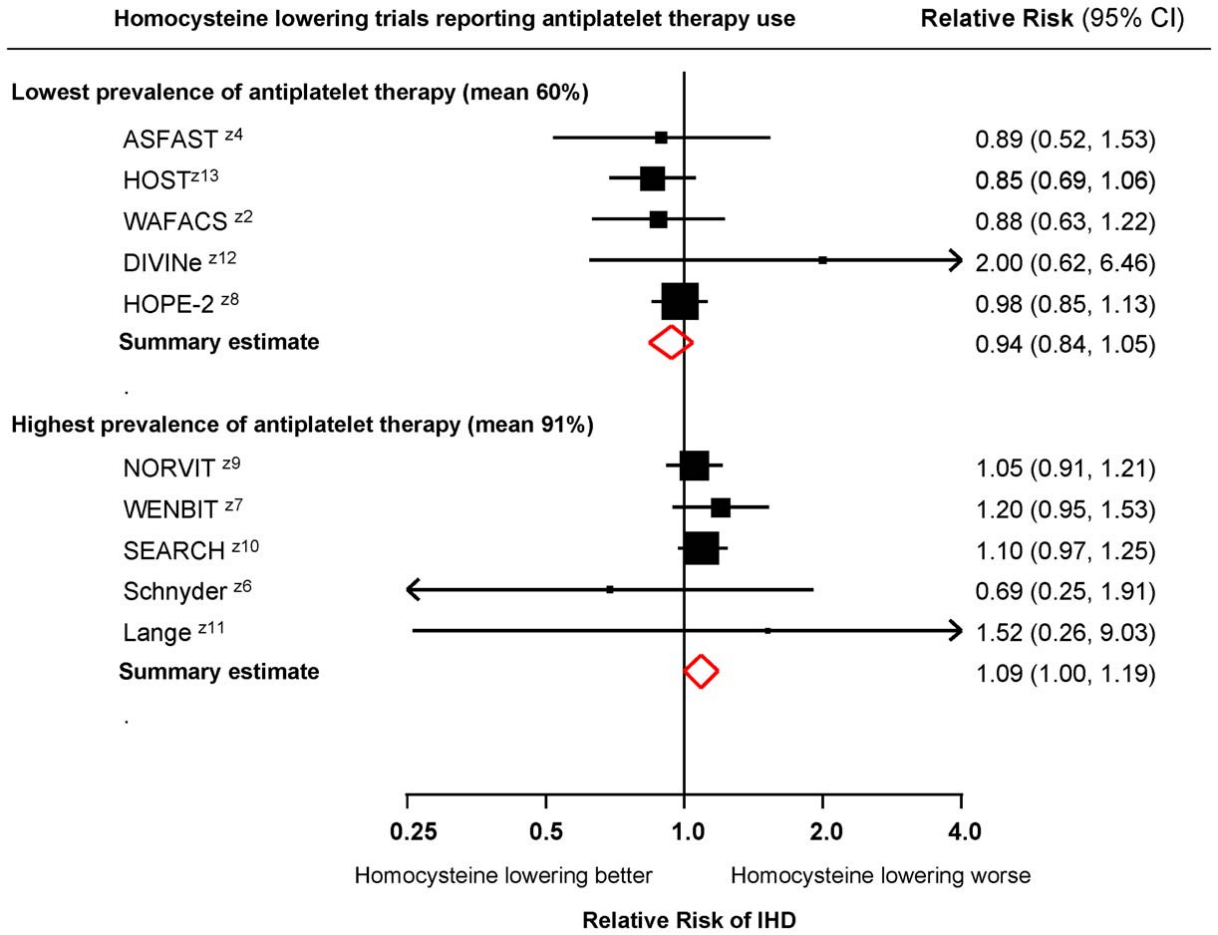
Meta-analysis of randomised trials of serum homocysteine reduction on ischaemic heart disease events (cardiac death and non-fatal myocardial infarction) according to use of antiplatelet therapy.

Figure 4 shows a meta-analysis plot of the 10 trials that reported the use of antiplatelet therapy, separated in to the 5 with the lowest prevalence of antiplatelet therapy (60% on average, usually aspirin) and the 5 with the highest prevalence (91% on average). The summary relative risks of ischaemic heart disease events were 0.94 (0.84 to 1.05) and 1.09 (1.00 to 1.19) respectively (p = 0.037 for the difference between the two estimates), suggesting an antiplatelet therapy-homocysteine interaction (see Discussion). A meta-regression, based on the individual trials, of the prevalence of anti-platelet therapy on relative risk of ischaemic heart disease gave a similar result (p = 0.056).

### Comparing the results from the two types of study

The meta-analysis of randomised trials showed that folic acid did not reduce the risk of ischaemic heart disease even though, on average, serum homocysteine was reduced by 3.3 µmol/L (relative risk (1.00 (0.93 to 1.08)). The meta-analysis of the MTHFR studies shows a statistically significant higher risk of IHD (odds ratio 1.16 (1.04 to 1.29) in TT than CC individuals for a 1.9 µmol/L homocysteine difference. This is equivalent to an odds ratio of 0.79 (0.67 to 0.93) for the same 3.3 µmol/L homocysteine difference observed in the trials. The summary result from the trials indicates that a reduction in risk of more than 7% (lower 95% confidence interval) is unlikely. The summary result from the MTHFR studies indicates that a reduction in risk of less than 7% (upper 95% confidence interval), for the same homocysteine difference, is unlikely. The results of the randomised trials and the MTHFR studies are thus discordant.

## Discussion

The discordant results from the randomised trials and MTHFR studies needs to be explained. A possible explanation is that lowering homocysteine may not add to the effect of aspirin and possibly other antiplatelet drugs in preventing ischaemic heart disease. Aspirin irreversibly blocks the formation of thromboxane A_2_ in platelets, producing an inhibitory effect on platelet activation, platelet aggregation and preventing thrombosis. [6] Homocysteine increases platelet activation, thromboxane production, and platelet aggregation; effects which have been demonstrated in animal and human studies and may explain the pathological effects of homocysteine in both arterial and venous disease. [7] A Medline literature search identified 22 studies on the effect of homocysteine on platelet function [8]–[29]. Seventeen studies showed a statistically significant (p<0.05) effect of homocysteine on increasing prothrombotc platelet function (increased platelet activation,[8]–[16] thromboxane production [17]–[20] or platelet aggregation[21]–[22]), 4 were inconclusive [25]–[28] and 1 showed an effect in the opposite direction.[29] The evidence that homocysteine increases prothrombotic platelet function is thus persuasive. In one of these studies on patients with homocystinuria, urinary excretion of thromboxane B2 (a metabolite of thromboxane A_2_) was about 5-fold higher in patients compared with healthy controls; an effect that was reversed on giving aspirin (50 mg/day). [18]

Given that homocysteine exerts a thrombotic effect through its action on platelet function, concomitant treatment with aspirin (or possibly other ant-platelet drugs) could reduce or negate the antiplatelet effect of lowering homocysteine in the trials. All but 4 of the randomised trials were conducted in patients with prior vascular disease, most of whom took antiplatelet drugs (usually aspirin) during the trial. Aspirin use prior to a diagnosis of ischaemic heart disease in the MTHFR studies was not reported, but this is likely to have been rare in individuals without ischaemic heart disease, because antiplatelet drugs were not routinely used in the absence of a specific indication (such as aspirin for arthritis). A negative interaction between antiplatelet therapy and homocysteine would not, therefore, apply in the MTHFR studies.

The hypothesis that aspirin reduces or negates the anti-platelet effect of lowering homocysteine, receives support from the observation that there was a statistically significant difference in risk reduction between the 5 trials with the lowest prevalence of concomitant antiplatelet therapy and the 5 trials with highest prevalence (p = 0.037). A limitation of this analysis is that it could only be undertaken on 10 of the 14 trials and it is possible that the division of trials in to the two groups could have given rise to a chance finding. However the hypothesis was proposed before the analysis was performed (ie. it was not data-derived) and it would explain the discordance between the results of the randomised trials and the MTHFR studies. Another limitation is that data were not available to explore the possibility, albeit unlikely, that individuals who took antiplatelet therapy in the trials, had an underlying medical condition that negated a preventive effect of homocysteine lowering on ischaemic heart disease.

The 5 trials with the lowest prevalence of antiplatelet therapy yielded a relative risk of ischaemic heart disease of 0.94. If the proposed hypothesis is correct, this relative risk estimate would arise only from the 40% of trial participants who were not taking concomitant aspirin, so the expected relative risk (RR) if none were taking aspirin would be 0.85 (0.4× RR +0.6×1 = 0.94); an estimated 15% reduction in risk of ischaemic heart disease. The fact that most of the trials included patients with pre-existing cardiovascular disease leaves open the possibility that folic acid has a useful role in the primary prevention of ischaemic heart disease, where aspirin is generally not used, but not in secondary prevention, where it is routine.

### Weighing the evidence

The evidence from the MTHFR studies indicates an association that is unlikely to be due to chance (p = 0.006). The studies exclude confounding, as generally understood, because the raised homocysteine concentrations occur as a result of a genetic mutation randomly distributed across the population; people with and without the mutation would not in expectation differ in other cardiovascular risk factors and direct observation has shown that they do not.[3] Genetic confounding is theoretically possible if there were a gene linked to the MTHFR polymorphism that also increases serum homocysteine and IHD risk. No such gene linkage has been identified and the probability of it accounting for the positive associations linking the MTHFR variant, homocysteine and ischaemic heart disease is, as previously described, low.[3], [30] The studies are, in effect, natural randomised experiments, capable of testing whether moderately raised homocysteine causes ischaemic heart disease. The result supports a causal relationship. A previous meta-analysis of MTHFR studies and stroke found a statistically significant effect (odds ratio 1.26 (1.14 to 1.40) for TT versus CC) and the authors concluded that the association was causal. [30]

The finding of significant heterogeneity (greater variation between study results than would be expected through chance) in the MTHFR studies is expected because the expression of the mutation on serum homocysteine levels depends on environmental factors, notably dietary folate. So, if dietary folate is high, the expression of the TT phenotype is reduced, homocysteine is less elevated and therefore the risk of ischaemic heart disease is less increased. [1], [3], [30,]. There were few direct measures of dietary folate intake in the studies available, so stratified analyses according to folate intake were not possible. The closest one can get to explaining the heterogeneity is the meta-regression illustrated in Figure 2, and this indicates that studies in populations with similar homocysteine in TT and CC groups tended to find no difference in ischaemic heart disease risk, whereas studies in populations with higher homocysteine in TT than CC groups tended to find higher risk (p = 0.013). However the limitations in our ability to explain the heterogeneity do not invalidate the result that, in general, TT individuals have a higher ischaemic heart disease risk than CC individuals.

Publication bias (the preferential publication of small positive studies over small negative ones) is likely to influence the meta-analysis but not to an extent that would explain the overall positive result. From Figure 1, 12 out of the 75 MTHFR studies had statistically significantly positive results but only two were statistically significantly negative. If there were no true association between the MTHFR polymorphism and ischaemic heart disease risk then the probability of a statistically significant result arising by chance (at the 5% level) would be 1 in 20 or 1 in 40 for positive results and 1 in 40 for negative results. From the 75 studies, about two statistically significantly positive results and two statistically significantly negative results would be expected by chance. The two statistically significant negative results are therefore expected but the 12 statistically significantly positive results are not (p = 0.01 for the difference between the two expected and 12 observed). For publication bias to have generated the positive overall result, therefore, these 12 studies would have to have come from a pool of 480 (12×40) studies, with 405 (480-75) of them remaining unpublished. It is unlikely that so large a number of researchers would fail to publish their studies, effectively excluding publication bias as a plausible explanation for the positive result.

In assessing whether homocysteine causes ischaemic heart disease, the effect of lowering serum homocysteine in reducing cardiovascular disease risk in patients with homocystinuria is relevant. Individuals with homozygous homocystinuria have homocysteine concentrations about 5 times greater than average and a 1 in 2 chance of a vascular disease event before age 30. [31] Treatment to lower serum homocysteine reduced risk by about 90% (compared with the risk in non-randomised untreated controls), an effect so large that selection bias is unlikely to account for the result. [32], [33] This supports the causal conclusion from the MTHFR studies.

Although the meta-analysis of trials on ischaemic heart disease showed no protective effect of homocysteine reduction, a meta-analysis of trials of serum homocysteine reduction on stroke prevention by Wang and colleagues showed that folic acid reduced the risk of stroke by 18% (relative risk 0.82 (0.68–1.00)). [34] It would be surprising to observe a preventive effect on stroke but not on ischaemic heart disease. Four of the eight trials in Wang's meta-analysis included patients with renal disease or oesophageal dysplasia and would not tend to be on antiplatelet therapy. This may explain the observed preventive effect of folic acid in the stroke trial meta-analysis and not in the ischaemic heart disease meta-analysis.

In conclusion, the negative trial evidence on ischaemic heart disease should not trump the positive evidence from the MTHFR studies and hence mistakenly lead to a conclusion that there is no role for folic acid in preventing ischaemic heart disease. There is evidence that folic acid has a modest but useful role in the primary prevention of ischaemic heart disease and concomitant use of aspirin, and possibly other antiplatelet therapy, is an explanation for why this is not evident in the results of the randomised trials.

## Supporting Information

Figure S1 **Screening and selection of studies flow chart.**
(TIF)

Figure S2 **Plot of the standard error (log odds ratio TT versus CC) against the odds ratio for each MTHFR study (dotted lines are 95% confidence limits) providing evidence against publication bias explaining the results.**
(TIF)

Table S1 **Citations of published articles providing information for meta-analysis of MTHFR studies comparing the prevalence of TT with CC homozygotes in cases with ischaemic heart disease and controls (** **Figure 1** **).**
(DOC)

Table S2 **Studies reporting serum homocysteine (µmol/L) according to MTHFR genotype in people without cardiovascular disease.**
(DOC)

Table S3 **Citations of published articles reporting serum homocysteine according to MTHFR genotype in people without a history of cardiovascular disease (Table S2).**
(DOC)

Table S4 **Citations of published randomised trials of B vitamins and ischaemic heart disease events included in the Meta-analysis (** **Figure 3** **).**
(DOC)
