# Supplementary figures and images for: Reconciling the Evidence on Serum Homocysteine and Ischaemic Heart Disease: A Meta-Analysis
Source: PLoS One. 2011 Feb 2;6(2):e16473. doi: 10.1371/journal.pone.0016473 (PMC3032783; doi:10.1371/journal.pone.0016473)

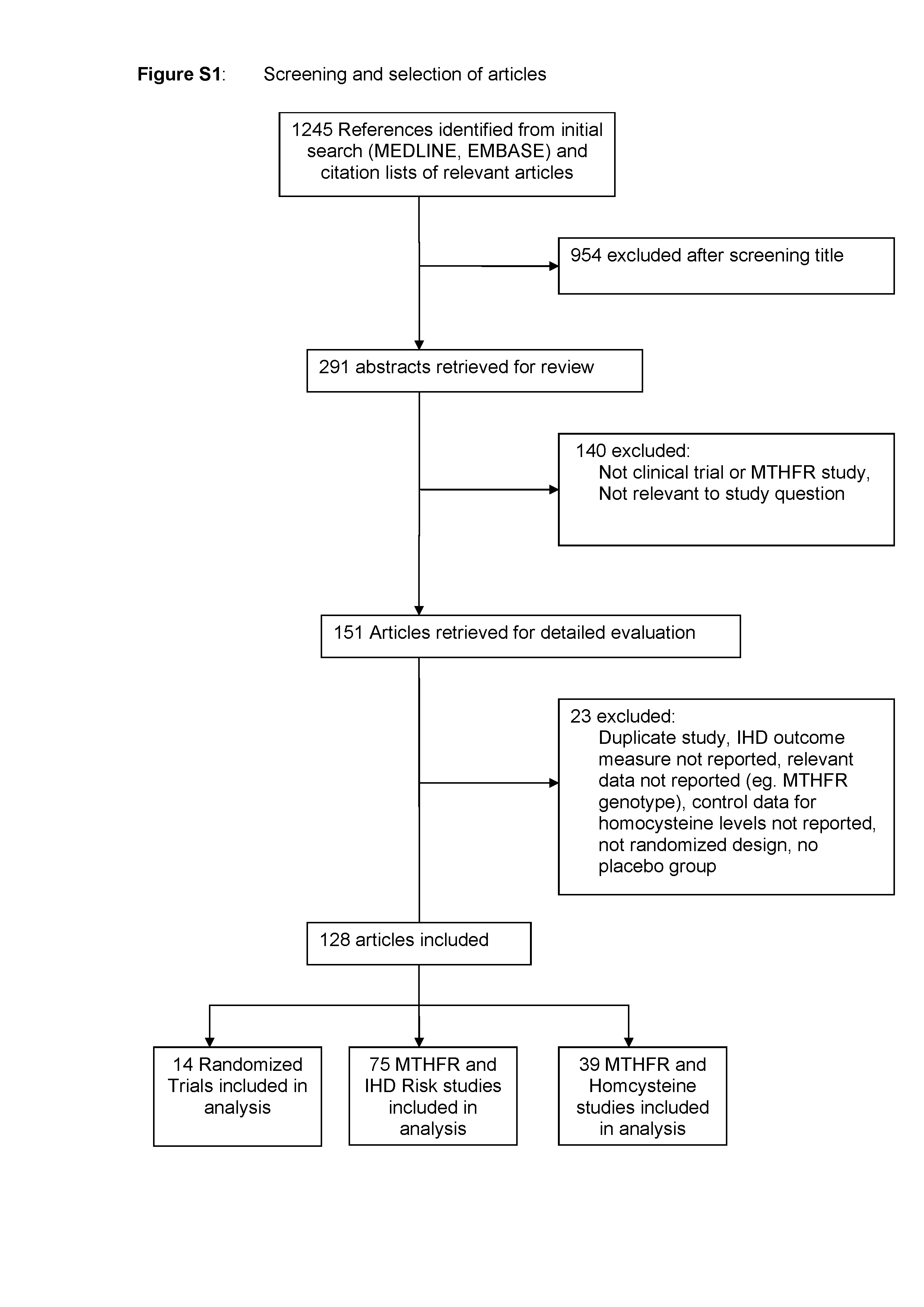

Supplement: Figure S1 — Screening and selection of studies flow chart. (TIF) [file pone.0016473.s001.tif]

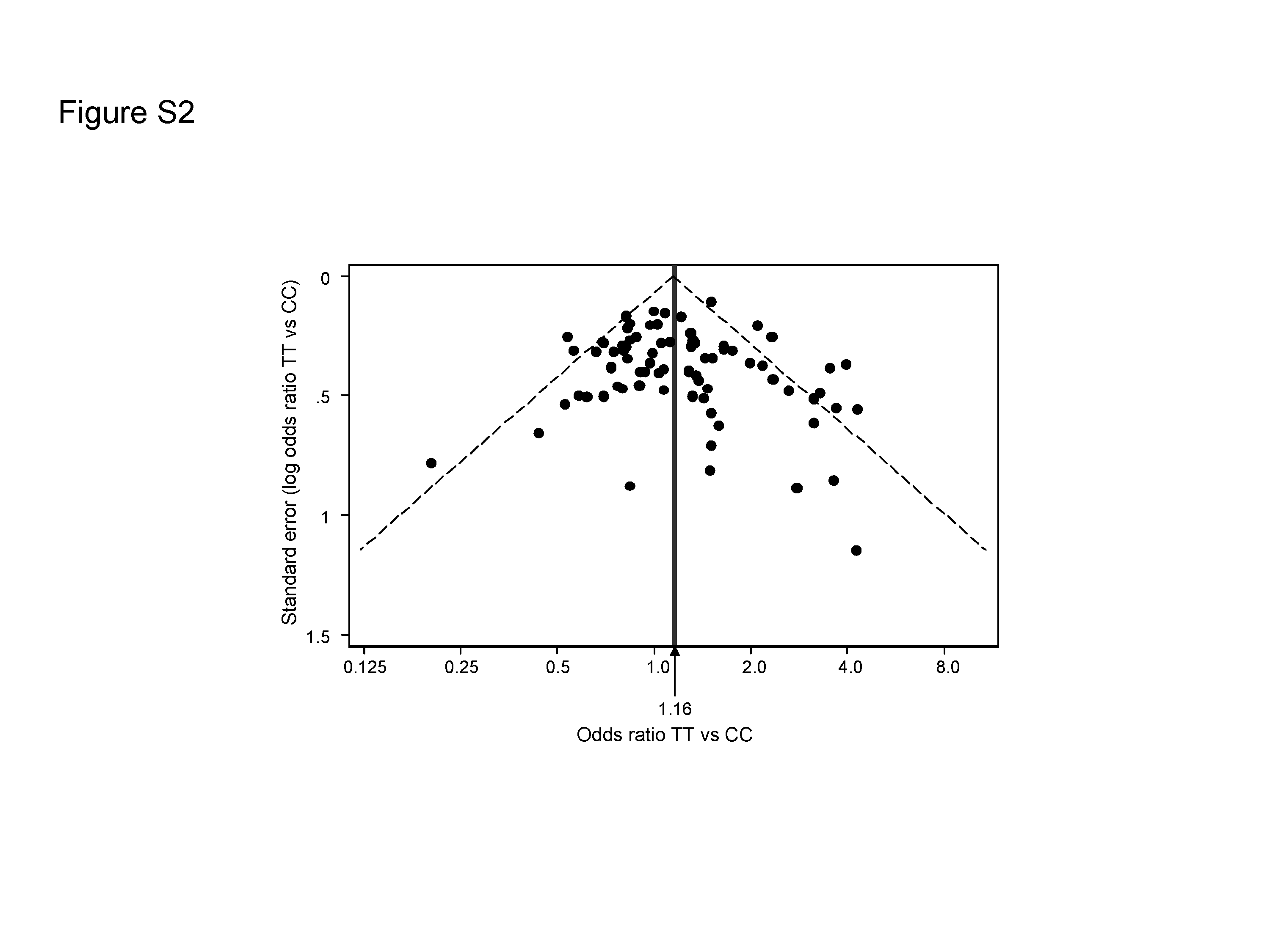

Supplement: Figure S2 — Plot of the standard error (log odds ratio TT versus CC) against the odds ratio for each MTHFR study (dotted lines are 95% confidence limits) providing evidence against publication bias explaining the results. (TIF) [file pone.0016473.s002.tif]
