# Supplementary material for: Reconciling the Evidence on Serum Homocysteine and Ischaemic Heart Disease: A Meta-Analysis
Source: PLoS One. 2011 Feb 2;6(2):e16473. doi: 10.1371/journal.pone.0016473 (PMC3032783; doi:10.1371/journal.pone.0016473)
Supplement: Table S1 — Citations of published articles providing information for meta-analysis of MTHFR studies comparing the prevalence of TT with CC homozygotes in cases with ischaemic heart disease and controls ( Figure 1 ). (DOC) [file pone.0016473.s003.doc]

**Table S1**: Citations of published articles providing information for meta-analysis of MTHFR studies comparing the prevalence of TT with CC homozygotes in cases with ischaemic heart disease and controls (Figure 1).

1. Zheng YZ, Tong J, Do XP, Pu XQ, Zhou BT (2000) Prevalence of methylenetetrahydrofolate reductase C667T and its association with arterial and venous thrombosis in the Chinese population. *Br J Haematol* 109:870-4.
2. Chambers JC, Ireland H, Thompson E, Reilly P, Obeid OA, Refsum H, et al. (2000) Methylenetetrahydrofolate reductase 677 C T mutation and coronary heart disease in UK Indian Asians. *Arterioscler Thromb Vasc Biol* 20:2448-52.
3. Fernandez-Arcas N, Diegues-Lucena JL, Munoz-Moran E, Ruiz-Galdon M, Espinosa-Caliani S, Aranda-Lara P, et al. (1999) The genotype interactions of methylenetetrahydrofolate reductase and renin-angiotensin system genes are associated with myocardial infarction. *Atherosclerosis* 145:293-300.
4. Meleady R, Ueland PM, Blom H. (2003) Thermolabile methylenetetrahydrofolate reductase, homocysteine, and cardiovascular disease risk: the European Concerted Action Project. *Am J Clin Nutr* 77(1):63-70.
5. Verhoeff BJ, Trip MD, Prins MH. (1998) The effect of a common methylenetetrahydrofolate reductase mutation on levels of homocysteine, folate, vitamin B12 and on the risk of premature atherosclerosis. *Atherosclerosis* 141(1):161-166
6. Zhang G, Dai C (2001) Gene polymorphisms of homocysteine metabolism-related enzymes in Chinese patients with occlusive coronary artery or cerebral vascular diseases. *Thromb Res* 104(3):187-95.
7. Chao CL, Tsai HH, Lee CM. (1999) The graded effect of hyperhomocysteinemia on the severity and extent of coronary atherosclerosis.*Atherosclerosis* 147(2):379-86.
8. Jang Y, Park HY, Lee JH, Ryu HJ, Kim JY et al.(2002) A polymorphism of the methylenetetrahydrofolate reductase and methionine synthase gene in CAD patients: association with plasma folate, vitamin B12 and homocysteine. *Nutrition Research* 22(9):965-76.
9. Brulhart MC, Dussoix P, Ruiz J, Passa P, Froguel P et al.(1997) The (Ala-Val) mutation of methylenetetrahydrofolate reductase as a genetic risk factor for vascular disease in non-insulin-dependent diabetic patients. *Am J Hum Genet* 60:228-9.
10. Gorqcy I, Gorcqy J, Suliga M, Ciechanowicz A (1999) C677T gene polymorphism of methelenetetrahydrofolate reductase (MTHFR) in patients with myocardial infarction. *Polskie Archivum Medycyny Wewnetrzaej* 4(10):849-54.
11. Roest M, van der Schouw YT, Grobbee DE. (2001) Methylenetetrahydrofolate reductase 677 C/T genotype and cardiovascular disease mortality in postmenopausal women. *Am J Epidemiol* 153(7):673-9.
12. Tsai MY, Welge BG, Hanson NQ, Bignell MK, Vessey J et al. (1999) Genetic causes of mild hyperhomocysteinemia in patients with premature occlusive coronary artery diseases. *Atherosclerosis* 143:163-70.
13. Girelli D, Friso S, Trabetti E, Olivieri O, Russo C et al. (1998) Methylenetetrahydrofolate reductase C677 mutation, plasma homocysteine, and folate in subjects from northern Italy with and without severe coronary atherosclerotic disease: evidence for an important genetic-environment interaction. *Blood* 91:4158-63.
14. Brugada R, Marian AJ (1997) A common mutation in methylenetetrahydrofolate reductase gene is not a major risk of coronary artery disease or myocardial infarction. *Atherosclerosis* 128:107-12.
15. Kadziela J, Janas J, Dzielinska Z. (2003) The C677T mutation in methylenetetrahydrofolate reductase gene, plasma homocysteine concentration and the risk of coronary artery disease. *Kardiol Pol* 59(7):17-26; discussion 26.
16. Adams M, Smith PD, Martin D, Thomson JR, Lodwick D et al.(1996) Genetic analysis of thermolabile methylenetetrahydrofolate reductase as a risk factor for myocardial infarction. *Q J Med* 89:437-44.
17. Schmitz C, Lindpainter K, Verhoef P, Gaziano JM, Buring J (1996) Genetic polymorphism of methylenetetrahydrofolate reductase and myocardial infarction: a case-control study. *Circulation* 94:1812-14.
18. Ardissino D, Mannucci PM, Merlini PA, Duca F, Fetiveau R et al. (1999) Prothrombotic genetic risk factors in young survivors of myocardial infarction. *Blood* 94:46-51.
19. Meisel C, Cascorbi I, Gerloff T, Stangl V, Laule M et al. (2001) Identification of six methylenetetrahydrofolate reductase (MTHFR) genotypes resulting from common polymorphisms: impact on plasma homocysteine levels and development of coronary artery disease. *Atherosclerosis* 154:651-8.
20. Hanson NQ, Aras O, Yang F, Tsai MY (2001) C677T and A1298C polymorphisms of the methylenetetrahydrofolate reductase gene: incidence and effect of combined genotypes on plasma fasting and post-methionine load homocysteine in vascular disease. *Clin Chem* 47(4):661-6.
21. Brilakis ES, Berger PB, Ballman KV, Rozen R (2003) Methylenetetrahydrofolate reductase (MTHFR) 677C>T and methionine synthase reductase (MTRR) 66A>G polymorphisms: association with serum homocysteine and angiographic coronary artery disease in the era of flour products fortified with folic acid. *Atherosclerosis* 168(2):315-22.
22. Verhoef P, Rimm EB, Hunter DJ, Chen J, Willett WC et al. (1998) A common mutation in the methylenetetrahydrofolate reductase gene and risk of coronary heart disease: results among US men. *J Am Coll Cardiol* 32:253-9.
23. Ma J, Stampfer MJ, Hennekens CH, Frosst P, Selhub J et al. (1996) Methylenetetrahydrofolate reductase polymorphism, plasma folate, homocysteine and risk of myocardial infarction in US physicians. *Circulation* 94:2410-6.
24. Dilley A, Hooper WC, El-Jamil M, Renshaw M, Wenger NK et al. (2001) Mutations in the genes regulating methylenetetrahydrofolate reductase (MTHFR C T677) and cystathione β -synthase (CBS G A919, CBS T c833) are not associated with myocardial infarction in African Americans. *Thromb Res* 103:109-15.
25. Rothenbacher D, Fischer HG, Hoffmeister A. (2002) Homocysteine and methylenetetrahydrofolate reductase genotype: association with risk of coronary heart disease and relation to inflammatory, hemostatic, and lipid parameters. *Atherosclerosis* 162(1):193-200.
26. Schwartz SM, Siscovick DS, Malinow MR, Rosendaal FR, Beverly RK et al. (1997) Myocardial infarction in young women in relation to plasma total homocysteine, folate, and a common variant in the methylenetetrahydrofolate reductase gene. *Circulation* 96:412-7.
27. Hsu L-A, Jo Y-L, Wand S-M, Chang C-J, Hsu T-S et al. (2001) The C677T mutation of the methylenetetrahydrofolate reductase gene is not associated with the risk of coronary artery disease or venous thrombosis among Chinese in Taiwan. *Hum Hered* 51:41-5.
28. Rossi GP, Maiolino G, Seccia TM. (2006) Hyperhomocysteinemia predicts total and cardiovascular mortality in high-risk women. *J Hypertens* 24(5):851-9.
29. Hong SH, Song J, Kim JQ (2001) Genetic variation of the methylenetetrahydrofolate reductase and cystathionine beta-synthase genes in Korean patients with coronary artery disease and a new polymorphism in intron 7. *Mol Cell Probes* 15(2):119-23.
30. Tobin MD, Braund PS, Burton PR. (2004) Genotypes and haplotypes predisposing to myocardial infarction: a multilocus case-control study. *Eur Heart J* 25(6):459-67.
31. van Bockxmeer FM, Mamotte CDS, Vasikaran SD, Taylor RR (1997) Methylenetetrahydrofolate reductase gene and coronary artery disease. *Circulation* 95:21-3.
32. Shioji K, Kokubo Y, Goto Y, Nonogi H, Iwai N (2004) An association analysis between genetic polymorphisms of matrix metalloproteinase-3 and methylenetetrahydrofolate reductase and myocardial infarction in Japanese. *J Thromb Haemost* 2(3):527-8.
33. Benes P, Kankova K, Muzik J. (2001) Methylenetetrahydrofolate reductase polymorphism, type II diabetes mellitus, coronary artery disease, and essential hypertension in the Czech population. *Mol Genet Metab* 73(2):188-95.
34. Abbate R, Sardi I, Pepe G, Marcucci R, Brunelli T et al. (1998) The high prevalence of the thermolabile 5,10-methylenetetrahydrofolate reductase (MTHFR) in Italians is not associated with an increased risk for coronary artery disease (CAD). *Thromb Haemost* 79:727-30.
35. Wilcken DEL, Wang XL, Sim AS, McCredie M (1996) Distribution in healthy and coronary populations of the methylenetetrahydrofolate reductase (MTHFR) C677T mutation. *Arterioscler Thromb Vasc Biol* 16:878-82.
36. Pintó X, Vilaseca MA, Garcia-Giralt N, Ferrer I, Palá M et al. (2001) Homocysteine and the MTHFR 677C T allele in premature coronary artery disease. Case control and family studies. *Eur J Clin Invest* 31:24-30.
37. Kim C-H, Hwang K-Y, Choi T-M, Shin W-Y, Hong S-Y (2001) The methylenetetrahydrofolate reductase gene polymorphism in Koreans with coronary artery disease. *Int J Cardiol* 78:13-17.
38. Kolling K, Ndrepepa G, Koch W. (2004) Methylenetetrahydrofolate reductase gene C677T and A1298C polymorphisms, plasma homocysteine, folate, and vitamin B12 levels and the extent of coronary artery disease. *Am J Cardiol* 93(10):1201-6.
39. Anderson JL, King GJ, Thomson MJ, Todd M, Bair TL et al. (1997) A mutation in the methylenetetrahydrofolate reductase gene is not associated with increased risk for coronary artery disease or myocardial infarction. *J Am Coll Cardiol* 30:1206-11.
40. Kluijtmans LAJ, Kastelein JJP, Lindemans J, Boers GHJ, Heil SG et al. (1997) Thermolabile methylenetetrahydrofolate reductase in coronary artery disease. *Circulation* 96:2573-7.
41. Fowkes FG, Lee AJ, Hau CM, Cooke A, Connor JM et al. (2000) Methylenetetrahydrofolate reductase (MTHFR) and nitric oxide synthase (ecNOS) genes and risks of peripheral and arterial disease and coronary heart disease: Edinburgh Artery Study. *Atherosclerosis* 150:179-85.
42. Christensen B, Frosst P, Lussier-Cacan S, Selhub J, Goyette P et al. (1997) Correlation of a common mutation in the methylenetetrahydrofolate reductase gene with plasma homocysteine in patients with premature coronary artery disease. *Arterioscler Thromb Vasc Biol* 17:569-73.
43. Topol EJ, McCarthy J, Gabriel S. (2001) Single nucleotide polymorphisms in multiple novel thrombospondin genes may be associated with familial premature myocardial infarction. *Circulation* 104(22):2641-4.
44. Gardemann A, Weidemann H, Philpp M, Katz N, Tillmanns H, Herlein F, et al. (1999) The TT genotype of the methylenetetrahydrofolate reductase C677T gene polymorphism is associated with the extent of coronary atherosclerosis in patients at high risk of coronary artery disease. *Eur Heart J* 20:584-92.
45. Spiridonova MG, Stepanov VA, Pyzyrev VP, Karpov RS (2000) [Relationship between polymorphism C677T of the methylene tetrahydrofolate reductase gene with clinical symptoms of coronary atherosclerosis]. *Genetika* 36(9):1269-73.
46. Tanis BC, Blom HJ, Bloemenkamp DG. (2004) Folate, homocysteine levels, methylenetetrahydrofolate reductase (MTHFR) 677C --> T variant, and the risk of myocardial infarction in young women: effect of female hormones on homocysteine levels. *J Thromb Haemost* 2(1):35-41.
47. Todesco L, Angst C, Litynski P, Loehrer F, Fowler B et al. (1999) Methylenetetrahydrofolate reductase polymorphism, plasma homocysteine and age. *Eur J Clin Invest* 29:1003-9.
48. Reinhardt D, Sigush HH, Vogt SF, Farker K, Muller S et al. (1998) Absence of association between common mutation in the methylenetetrahydrofolate reductase gene and the risk of coronary artery disease. *Eur J Clin Invest* 28:20-3.
49. Verhoef P, Kok FJ, Kluijtmans LAJ, Blom HJ, Refsum H et al. (1997) The 677C T mutation in the methylenetetrahydrofolate reductase gene: associations with plasma total homocysteine levels and risk for coronary atherosclerotic disease. *Atherosclerosis* 132:105-13.
50. Zuntar I, Topic E, Vukosavic D. (2003) Croatian population data for the C677T polymorphism in methylenetetrahydrofolate reductase: frequencies in healthy and atherosclerotic study groups. *Clin Chim Acta* 335(1-2):95-100.
51. Kihara T, Abe S, Saigo M, Kaieda H, Obata H et al. (1997) Methylenetetrahydrofolate reductase gene polymorphism and premature myocardial infarction. *Circulation* 96:101-I.
52. Araujo F, Lopes M, Goncalves L, Maiciel MJ, Cunha-Ribeiro LM (2000) Hyperhomocysteinemia, MTHFR C667T genotype and low folate levels: a risk combination for acute coronary disease in a Portuguese population. *Thromb Haemost* 83:517-8.
53. Tokgozoglu SL, Alikasifoglu M, Unsal I, Atalar E, Aytemir K et al. (1999) Methylenetetrahydrofolate reductase genotype and the risk and extent of coronary artery disease in a population with low plasma folate. *Heart* 81:518-22.
54. Yamada Y, Matsuo H, Segawa T. (2006) Assessment of genetic risk formyocardial infarction. *Thromb Haemost* 96(2):220-7.
55. Chen TY, Chen JH, Tsao CJ (2000) Methylenetetrahydrofolate reductase gene polymorphism and coronary artery disease in Taiwan Chinese. *Haematologica* 85(4):445-6.
56. Malik NM, Syrris P, Schwartsman R, Kaski JC, Crossman DC et al. (1998)Methylenetetrahydrofolate reductase polymorphism (C-677T) and coronary artery disease. *Clin Sci* 95:311-5.
57. Thögersen AM, Nilsson TK, Dahlen G, Jansson J-H, Boman K et al. (2001) Homozygosity for the C^677^ T mutation of 5,10-methylenetetrahydrofolate reductase and total plasma homocyst(e)ine are not associated with greater than normal risk of a first myocardial infarction in northern Sweden. *Coron Artery Dis* 12:85-90.
58. Izumi M, Iwai N, Ohmichi N, Nakamura Y, Shimoike H et al. (1996) Molecular variant of 5,10-methylenetetrahydrofolate reductase is a risk factor of ischaemic heart disease in the Japanese population. *Atherosclerosis* 121:293-4.
59. Nakai K, Fusazaki T, Suzuki T, Ohsawa M, Kamata J et al. (2000) Genetic polymorphism of 5,10-methylenetetrahydrofolate increases risk of myocardial infarction and is correlated to elevated levels of homocysteine in the Japanese general population. *Coron Artery Dis* 11:47-51.
60. Abu-Amero KK, Wyngaard CA, Dzimiri N (2003) Prevalence and role of methylenetetrahydrofolate reductase 677 C-->T and 1298 A-->C polymorphisms in coronary artery disease in Arabs. *Arch Pathol Lab Med* 127(10):1349-52.
61. Morita H, Taguchi J, Kurihara H, Kitaoka M, Kaneda H et al. (1997) Genetic polymorphism of 5,10-methylenetetrahydrofolate reductase (MTHFR) as a risk factor for coronary artery disease. *Circulation* 95:2032-6.
62. Szczeklik A, Sanak M, Jankowski M, Dropinski J, Czachór R et al. (2001) Mutation A1298C of methylenetetrahydrofolate reductase: risk for early coronary disease not associated with hyperhomocysteinemia. *Am J Med Genet* 101:36-9.
63. Ou T, Kobayashi Y, Yamakawa K, Arinami T, Amemiya H et al. (1998) Methylenetetrahydrofolate reductase and apolipoprotein E polymorphisms are independent risk factors for coronary heart disease in Japanese: a case control study. *Atherosclerosis* 137:23-8.
64. Sarecka-Hujar B, Zak I, Krauze J (2008) Carrier-state of two or three polymorphic variants of MTHFR, IL-6 and ICAM1 genes increases the risk of coronary artery disease. *Kardiol Pol* 66(12):1269-77.
65. Malinow MR, Nieto FJ, Kruger WD, Duell PB, Hess DL et al. (1997) The effects of folic acid supplementation on plasma total homocysteine are modulated by multivitamin use and methylenetetrahydrofolate reductase genotypes. *Arterioscler* *Thromb Vasc Biol* 17:1157-62.
66. Ilhan N, Kucuksu M, Kaman D, Ilhan N, Ozbay Y (2008) The 677 C/T MTHFR polymorphism is associated with essential hypertension, coronary artery disease, and higher homocysteine levels. *Arch Med Res* 39(1):125-30.
67. Kawashiri M, Kajinami K, Nohara A, Yagi K, Inazu A et al. (2000) Effect of common methylenetetrahydrofolate reductase gene mutation on coronary artery disease in familial hypercholesterolaemia. *Am J Cardiol* 86:840-5.
68. Raslova K, Smolkova B, Vohnout B, Gasparovic J, Frohlich JJ (2001) Risk factors for atherosclerosis in survivors of myocardial infarction and their spouses: comparison to controls without personal and family history of atherosclerosis. *Metabolism* 50(1):24-9.
69. Gallagher PM, Meleady R, Shields DC, Soon Tan K, McMaster D et al. (1996) Homocysteine and risk of premature coronary heart disease: evidence for a common gene mutation. *Circulation* 94:2154-8.
70. Mager A, Lalezari S, Shohat T, Birnbaum Y, Adler Y et al. (1999) Methylenetetrahydrofolate reductase genotypes and early onset coronary artery disease. *Circulation* 100:2406-10.
71. Kou MC, Wang L, Liang KJ, Wu M. Genotyping 5,10-methylenetetrahydrofolate reductase for patients with coronary artery disease in southern Taiwan. *Journal of Food and Drug Analysis* 9:173-7.
72. Ferrer-Antunes C, Palmeiro A, Morais J, Lourenço M, Freitas M et al. (1998) The mutation C677T in the methylenetetrahydrofolate reductase gene as a risk factor for myocardial infarction in the Portuguese population. *Thromb Haemost* 80:521-2.
73. Inbal A, Freimark D, Modan B. (1999) Synergistic effects of prothrombotic polymorphisms and atherogenic factors on the risk of myocardial infarction in young males. *Blood* 93(7):2186-90.
74. Payne DA, Chamoun AJ, Seifert SL, Stouffer GA (2001) MTHFR 677 C-->T mutation: a predictor of early-onset coronary artery disease risk. *Thromb Res* 103(4):275-9.
75. Güleç S, Aras Ö, Akar E, Tutar E, Ömürlü K et al. (2001) Methylenetetrahydrofolate reductase gene polymorphism and risk of premature myocardial infarction. *Clin Cardiol* 24:281-4.
