# Supplementary material for: Reconciling the Evidence on Serum Homocysteine and Ischaemic Heart Disease: A Meta-Analysis
Source: PLoS One. 2011 Feb 2;6(2):e16473. doi: 10.1371/journal.pone.0016473 (PMC3032783; doi:10.1371/journal.pone.0016473)
Supplement: Table S2 — Studies reporting serum homocysteine (µmol/L) according to MTHFR genotype in people without cardiovascular disease. (DOC) [file pone.0016473.s004.doc]

| **Table S2: Studies reporting serum homocysteine (µmol/L) according to MTHFR genotype in people without cardiovascular disease** | | | | | | | | | | | | | | | | | | | |
| --- | --- | --- | --- | --- | --- | --- | --- | --- | --- | --- | --- | --- | --- | --- | --- | --- | --- | --- | --- |
|  | | **TT** | | | | | **CT** | | | | | **CC** | | | | | **Homocysteine difference** | | |
| **Study and first author** | | **Mean** | | **SD** | **No of subjects** | | **Mean** | | **SD** | **No of subjects** | | **Mean** | **SD** | | **No of subjects** | | **(TT minus CC)** | | **SE** |
| Caudill^y1^ | | 4.2 | | 1.0 | 9 | | 5.7 | | 1.7 | 50 | | 5.5 | 1.8 | | 75 | | -1.3 | | 0.4 |
| Ilhan^y2^ | | 12.5 | | 5.2 | 2 | | 13.7 | | 5.6 | 26 | | 13.8 | 7.1 | | 72 | | -1.3 | | 3.8 |
| Thogerson^y3^ | | 11.6 | | 2.6 | 5 | | 11.6 | | 3.9 | 40 | | 12.5 | 6.0 | | 55 | | -0.9 | | 1.4 |
| Schmitzy4 | | 9.1 | | 2.3 | 14 | | 10.6 | | 3.8 | 46 | | 9.9 | 2.7 | | 67 | | -0.8 | | 0.7 |
| Chambers (Asian)^y5^ | | 10.4 | | 4.1 | 12 | | 10.4 | | 3.6 | 90 | | 11.0 | 4.1 | | 279 | | -0.6 | | 1.2 |
| Rassoul^y6^ | | 8.0 | | 1.3 | 5 | | 8.1 | | 1.5 | 17 | | 8.2 | 1.5 | | 29 | | -0.2 | | 0.6 |
| Fohr^y7^ | | 8.5 | | 2.5 | 21 | | 8.5 | | 2.5 | 75 | | 8.5 | 1.9 | | 64 | | 0.0 | | 0.6 |
| Guinotte^y8^ | | 5.4 | | 0.6 | 17 | | 5.9 | | 0.9 | 12 | | 5.3 | 0.9 | | 14 | | 0.1 | | 0.3 |
| Jacques^y9^ | | 10.4* | |  | 149 | | 9.6* | |  | 152 | | 9.8* |  | | 450 | | 0.6 | | 0.3 |
| Ho^y10^ | | 9.8 | | 7.1 | 69 | | 9.0 | | 4.9 | 164 | | 9.0 | 4.9 | | 212 | | 0.8 | | 0.9 |
| Dekou (women)^y11^ | | 11.1 | | 3.2 | 36 | | 10.7 | | 3.1 | 149 | | 10.2 | 2.9 | | 161 | | 0.9 | | 0.6 |
| Thuillier^y12^ | | 9.0 | | 4.4 | 10 | | 8.7 | | 3.1 | 32 | | 8.0 | 2.2 | | 27 | | 1.0 | | 1.5 |
| Meisel^y13^ | | 10.3 | | 4.5 | 177 | | 9.7 | | 3.6 | 421 | | 9.3 | 4.5 | | 265 | | 1.0 | | 0.4 |
| Voutilainen^y14^ | | 11.9 | | 3.0 | 11 | | 9.9 | | 2.0 | 57 | | 10.9 | 2.2 | | 100 | | 1.0 | | 0.9 |
| Somekawa^y15^ | | 11.0 | | 0.5 | 30 | | 10.7 | | 0.3 | 91 | | 10.0 | 0.3 | | 96 | | 1.0 | | 0.1 |
| Silsate^y16^ | | 9.1 | | 2.6 | 5 | | 8.0 | | 2.2 | 13 | | 8.0 | 1.6 | | 19 | | 1.1 | | 1.2 |
| Malinow^y17^ | | 10.3 | | 3.8 | 25 | | 9.4 | | 3.7 | 128 | | 9.1 | 3.5 | | 89 | | 1.2 | | 0.8 |
| Hustad^y18^ | | 11.2* | |  | 850 | | 10.4* | |  | 4299 | | 9.9* |  | | 5452 | | 1.3 | | 0.2 |
| Cappuccio (women)^y19^ | | 10.3 | | 3.2 | 39 | | 9.4 | |  | 206 | | 9.0 | 4.0 | | 506 | | 1.3 | | 0.5 |
| Zee^y20^ | | 12.5 | | 6.1 | 2773 | | 11.4 | | 4.9 | 10966 | | 11.1 | 4.3 | | 11229 | | 1.4 | | 0.1 |
| Mazza^y21^ | | 8.7 | | 3.0 | 30 | | 7.5 | | 1.8 | 53 | | 7.3 | 1.9 | | 47 | | 1.4 | | 0.6 |
| Chango^y22^ | | 11.2 | | 5.3 | 49 | | 9.5 | | 2.8 | 125 | | 9.8 | 2.7 | | 117 | | 1.4 | | 0.8 |
| Castro^y23^ | | 9.8† | |  | 12 | | 9.2† | |  | 54 | | 8.3† |  | | 51 | | 1.5 | | 0.9 |
| Kolling^y24^ | | 13.2 | | 5.7 | 68 | | 11.9 | | 4.1 | 283 | | 11.7 | 4.5 | | 266 | | 1.5 | | 0.7 |
| Chambers (European)^y5^ | | 11.6 | | 5.5 | 41 | | 10.1 | | 3.0 | 195 | | 10.1 | 2.9 | | 188 | | 1.5 | | 0.9 |
| Chango^y25^ | | 10.7 | | 5.3 | 12 | | 9.3 | | 3.3 | 25 | | 9.0 | 2.7 | | 29 | | 1.7 | | 1.6 |
| Ma^y26^ | | 12.0 | | 0.7 | 39 | | 10.8 | | 0.4 | 116 | | 10.1 | 0.4 | | 193 | | 1.9 | | 0.1 |
| Friedman^y27^ | | 11.7 | | 6.7 | 55 | | 9.8 | | 5.8 | 172 | | 9.8 | 3.9 | | 150 | | 1.9 | | 1.0 |
| Cappuccio^y19^ | | 13.2* | |  | 25 | | 11.6* | |  | 185 | | 11.3* |  | | 410 | | 1.9 | | 0.8 |
| Rothenbacher^y28^ | | 10.3 | |  | 50 | | 8.5 | |  | 210 | | 8.4 |  | | 219 | | 2.0 | | 0.7 |
| Husemoen^y29^ | | 9.8† | |  | 46 | | 8.2† | |  | 141 | | 7.8† |  | | 185 | | 2.0 | | 0.7 |
| Ordonez^y30^ | | 11.3 | | 4.6 | 33 | | 10.1 | | 6.6 | 135 | | 9.2 | 5.3 | | 139 | | 2.1 | | 0.9 |
| Dekou (men)^y11^ | | 14.1 | | 4.1 | 36 | | 11.9 | | 3.1 | 188 | | 12.0 | 3.5 | | 184 | | 2.1 | | 0.7 |
| Reyes-Engel^y31^ | | 12.5 | | 2.2 | 119 | | 10.6 | | 3.4 | 413 | | 10.3 | 3.9 | | 249 | | 2.2 | | 0.3 |
| Inamoto^y32^ | | 13.3* | |  | 520 | | 11.2* | |  | 1539 | | 11.0* |  | | 1189 | | 2.3 | | 0.2 |
| Devlin^y33^ | | 15.5 | | 0.5 | 121 | | 13.5 | | 0.2 | 432 | | 12.9 | 0.2 | | 488 | | 2.6 | | 0.0 |
| Saw^y34^ | | 12.3* | |  | 39 | | 10.0* | |  | 165 | | 9.7* |  | | 273 | | 2.6 | | 0.7 |
| Schwartz^y35^ | | 13.5 | | 7.0 | 43 | | 10.8 | | 3.9 | 141 | | 10.9 | 3.8 | | 154 | | 2.6 | | 1.1 |
| Christensen^y36^ | | 12.9 | | 4.7 | 8 | | 10.5 | | 4.2 | 47 | | 10.2 | 5.2 | | 31 | | 2.7 | | 1.9 |
| Hustad^y37^ | | 11.5* | |  | 37 | | 9.1* | |  | 182 | | 8.6* |  | | 204 | | 2.9 | | 0.8 |
| Meleady^y38^ | | 12.2 | | 1.5 | 81 | | 9.7 | | 2.1 | 314 | | 9.3 | 2.1 | | 352 | | 2.9 | | 0.2 |
| Frederiksen^y39^ | | 13.9 | | 9.2 | 315 | | 11.0 | | 4.2 | 1600 | | 10.9 | 4.4 | | 1804 | | 3.0 | | 0.5 |
| Nakai^y40^ | | 11.6 | | 5.6 | 63 | | 8.9 | | 4.1 | 191 | | 8.6 | 3.3 | | 174 | | 3.0 | | 0.7 |
| Kluitjmens^y41^ | | 15.3 | | 5.8 | 23 | | 13.1 | | 4.0 | 112 | | 12.3 | 2.7 | | 134 | | 3.0 | | 1.2 |
| Tanis^y42^ | | 14.9 | | 5.3 | 59 | | 12.4 | | 3.2 | 262 | | 11.6 | 2.7 | | 280 | | 3.3 | | 0.7 |
| Passaro^y43^ | | 12.7 | | 2.7 | 20 | | 10.5 | | 2.2 | 72 | | 9.3 | 1.6 | | 28 | | 3.4 | | 0.7 |
| Bathum^y44^ | | 11.0 | | 6.4 | 102 | | 7.8 | | 2.7 | 475 | | 7.4 | 2.1 | | 556 | | 3.6 | | 0.6 |
| Jee^y45^ | | 13.4 | | 0.6 | 76 | | 9.8 | | 0.4 | 184 | | 9.7 | 0.5 | | 135 | | 3.7 | | 0.1 |
| Pullin^y46^ | | 12.5 | | 5.7 | 42 | | 9.3 | | 2.5 | 42 | | 8.8 | 2.4 | | 42 | | 3.7 | | 1.0 |
| Litynski^y47^ | | 16.2 | | 5.8 | 20 | | NA | | NA | NA | | 12.1 | 2.5 | | 20 | | 4.1 | | 1.4 |
| Ou^y48^ | | 15.1 | | 6.0 | 27 | | 11.2 | | 1.9 | 19 | | 10.5 | 3.3 | | 39 | | 4.6 | | 1.3 |
| Jang^y49^ | | 13.5 | | 6.8 | 48 | | 9 | | 3.6 | 115 | | 8.9 | 3.8 | | 67 | | 4.6 | | 1.1 |
| Madonna^y50^ | | 15.0 | | 11.6 | 45 | | 9.9 | | 3.8 | 70 | | 9.7 | 3.9 | | 84 | | 5.3 | | 1.8 |
| Verhoef^y51^ | | 17.4* | |  | 7 | | 12.1* | |  | 48 | | 10.9* |  | | 45 | | 6.5 | | 1.8 |
| D'Angelo^y52^ | | 18.1 | | 14.1 | 39 | | 11.4 | | 3.8 | 98 | | 10.1 | 2.7 | | 45 | | 8.0 | | 2.3 |
| Zittoun^y53^ | | 19.9 | | 10.0 | 6 | | 12.1 | | 3.9 | 20 | | 11.4 | 2.9 | | 26 | | 8.5 | | 4.1 |
| **Combined** |  | |  | | |  | |  | |  |  | | |  | |  | | **1.9** | **0.15** |
| *geometric mean †median, SD (standard deviation), SE (standard error | | | | | | | | | | | | | |  | |  | |  |  |
