# Supplementary material for: Reconciling the Evidence on Serum Homocysteine and Ischaemic Heart Disease: A Meta-Analysis
Source: PLoS One. 2011 Feb 2;6(2):e16473. doi: 10.1371/journal.pone.0016473 (PMC3032783; doi:10.1371/journal.pone.0016473)
Supplement: Table S3 — Citations of published articles reporting serum homocysteine according to MTHFR genotype in people without a history of cardiovascular disease (Table S2). (DOC) [file pone.0016473.s005.doc]

**Table S3:** Citations of published articles reporting serum homocysteine according to MTHFR genotype in people without a history of cardiovascular disease (Table S2).

1. Caudill MA, Le T, Moonie SA, Esfahani ST, Cogger EA (2001) Folate status in women of childbearing age residing in Southern California after folic acid fortification. *J Am Coll Nutr* 20(2 Suppl):129-34.
2. Ilhan N, Kucuksu M, Kaman D, Ilhan N, Ozbay Y (2008) The 677 C/T MTHFR polymorphism is associated with essential hypertension, coronary artery disease, and higher homocysteine levels. *Arch Med Res* 39(1):125-30.
3. Thogersen AM, Nilsson TK, Dahlen G. (2001) Homozygosity for the C677-->T mutation of 5,10-methylenetetrahydrofolate reductase and total plasma homocyst(e) ine are not associated with greater than normal risk of a first myocardial infarction in northern Sweden. *Coron Artery Dis* 12(2):85-90.
4. Schmitz C, Lindpaintner K, Verhoef P, Gaziano JM, Buring J (1996) Genetic polymorphism of methylenetetrahydrofolate reductase and myocardial infarction. A case-control study. *Circulation* 94(8):1812-4.
5. Chambers JC, Ireland H, Thompson E. (2000) Methylenetetrahydrofolate reductase 677 C-->T mutation and coronary heart disease risk in UK Indian Asians. *Arterioscler Thromb Vasc Biol* 20(11):2448-52.
6. Rassoul F, Richter V, Janke C. (2000) Plasma homocysteine and lipprotein profile in patients with peripheral arterial occlusive disease. *Angiology* 51(3);189-196
7. Fohr IP, Prinz-Langenohl R, Bronstrup A. (2002) 5,10-Methylenetetrahydrofolate reductase genotype determines the plasma homocysteine-lowering effect of supplementation with 5-methyltetrahydrofolate or folic acid in healthy young women. *Am J Clin Nutr* 75(2):275-82.
8. Guinotte CL, Burns MG, Axume JA, Hata H, Urrutia TF et al. (2003) Methylenetetrahydrofolate reductase 677C-->T variant modulates folate status response to controlled folate intakes in young women. *J Nutr* 133(5):1272-80.
9. Jacques PF, Kalmbach R, Bagley PJ, Russo GT, Rogers G et al. (2002) The relationship between riboflavin and plasma total homocysteine in the Framingham Offspring cohort is influenced by folate status and the C677T transition in the methylenetetrahydrofolate reductase gene. *J Nutr* 132(2):283-8.
10. Ho CH (2000) The influence of age, sex, vitamin B(12), folate levels and methylenetetrahydrofolate reductase C677T genetic mutations on plasma homocysteine in the Chinese population. *Haematologica* 85(10):1051-4.
11. Dekou V, Whincup P, Papacosta O Ebrahim S, Lennon L et al. (2001) The effect of the C677T and A1298C polymorphisms in the methylenetetrahydrofolate reductase gene on homocysteine levels in elderly men and women from the British regional heart study. *Atherosclerosis* 154(3):659-66.
12. Thuillier L, Chadefaux-Vekemans B, Bonnefont JP et al. (1998) Does the polymorphism 677C-T of the 5,10-methylenetetrahydrofolate reductase gene contribute to homocysteine-related vascular disease? *J Inherit Metab Dis* 21(8):812-22.
13. Meisel C, Cascorbi I, Gerloff T, Stangl V, Laule M et al. (2001) Identification of six methylenetetrahydrofolate reductase (MTHFR) genotypes resulting from common polymorphisms: impact on plasma homocysteine levels and development of coronary artery disease. *Atherosclerosis* 154(3):651-8.
14. Voutilainen S, Lakka TA, Hamelahti P, Lehtimäki T, Poulsen HE et al. (2000) Plasma total homocysteine concentration and the risk of acute coronary events: the Kuopio Ischaemic Heart Disease Risk Factor Studytpdel. *J Int Med* 248:217-222
15. Somekawa Y, Kobayashi K, Tomura S, Aso T, Hamaguchi H (2002) Effects of hormone replacement therapy and methylenetetrahydrofolate reductase polymorphism on plasma folate and homocysteine levels in postmenopausal Japanese women. *Fertil Steril* 77(3):481-6.
16. Silaste ML, Rantala M, Sampi M, Alfthan G, Aro A, Kesaniemi YA (2001) Polymorphisms of key enzymes in homocysteine metabolism affect diet responsiveness of plasma homocysteine in healthy women. *J Nutr* 131(10):2643-7.
17. Malinow MR, Nieto FJ, Kruger WD, Duell PB, Hess DL et al. (1997) The effects of folic acid supplementation on plasma total homocysteine are modulated by multivitamin use and methylenetetrahydrofolate reductase genotypes. *Arterioscler Thromb Vasc Biol* 17(6):1157-62.
18. Hustad S, Midttun O, Schneede J, Vollset SE, Grotmol T, Ueland PM (2007) The methylenetetrahydrofolate reductase 677C-->T polymorphism as a modulator of a B vitamin network with major effects on homocysteine metabolism. *Am J Hum Genet* 80(5):846-55.
19. Cappuccio FP, Bell R, Perry IJ, Gilg J, Ueland PM et al. (2002) Homocysteine levels in men and women of different ethnic and cultural background living in England. *Atherosclerosis* 164(1):95-102.
20. Zee RY, Mora S, Cheng S et al.(2007) Homocysteine, 5,10-methylenetetrahydrofolate reductase 677C>T polymorphism, nutrient intake, and incident cardiovascular disease in 24,968 initially healthy women. *Clin Chem* 53(5):845-51.
21. Mazza A, Motti C, Nulli A, Marra G, Gnasso A et al. (2000) Lack of association between cartoid intima-media thickness and methylenetetrahydrofolate reductase gene polymorphism or serum homocysteine in non-insulin-dependent diabetes melliuts. *Metabolism* 49(6):718-723
22. Chango A, Potier De Courcy G, Boisson F, Guilland JC et al. (2000) 5,10-methylenetetrahydrofolate reductase common mutations, folate status and plasma homocysteine in healthy French adults of the Supplementation en Vitamines et Mineraux Antioxydants (SU.VI.MAX) cohort. *Br J Nutr* 84(6):891-6.
23. Castro R, Rivera I, Ravasco PM, Camilo E, Jakobs C et al. (2003) 5,10-Methylenetetrahydrofolate reductase 677C-->T and 1298A-->C mutations are genetic determinants of elevated homocysteine. *QJM* 96(4):297-303.
24. Kolling K, Ndrepepa G, Koch W, Braun S, Mehilli J et al. (2004) Methylenetetrahydrofolate reductase gene C677T and A1298C polymorphisms, plasma homocysteine, folate, and vitamin B12 levels and the extent of coronary artery disease. *Am J Cardiol* 93(10):1201-6.
25. Chango A, Boisson F, Barbe F, Quilliot D, Droesch S et al. (2000) The effect of 677C-->T and 1298A-->C mutations on plasma homocysteine and 5,10-methylenetetrahydrofolate reductase activity in healthy subjects. *Br J Nutr* 83(6):593-6.
26. Ma J, Stampfer MJ, Hennekens CH. (1996) Methylenetetrahydrofolate reductase polymorphism, plasma folate, homocysteine, and risk of myocardial infarction in US physicians. *Circulation* 94(10):2410-6.
27. Friedman G, Goldschmidt N, Friedlander Y, Ben-Yehuda A, Selhub J et al. (1999) A common mutation A1298C in human methylenetetrahydrofolate reductase gene: association with plasma total homocysteine and folate concentrations. *J Nutr* 129(9):1656-61.
28. Rothenbacher D, Fischer HG, Hoffmeister A, Hoffmann MM, März W et al. (2002) Homocysteine and methylenetetrahydrofolate reductase genotype: association with risk of coronary heart disease and relation to inflammatory, hemostatic, and lipid parameters. *Atherosclerosis* 162(1):193-200.
29. Husemoen LL, Toft U, Fenger M, Jorgensen T, Johansen N et al. (2006) The association between atopy and factors influencing folate metabolism: is low folate status causally related to the development of atopy? *Int J Epidemiol* 35(4):954-61.
30. Ordonez AJG, Alvarez CRF, Rodriguez JMM, Garcia EC, Alvarez MV. (1999) Genetic polymorphism of methylenetetrahydrofolate reductase and venous thromboembolism : a case-control study. *Haematologica* Feb;84(2):190-191
31. Reyes-Engel A, Munoz E, Gaitan MJ, Fabre Em Gallo M et al. (2002) Implications on human fertility of the 677C-->T and 1298A-->C polymorphisms of the MTHFR gene: consequences of a possible genetic selection. *Mol Hum Reprod* 8(10):952-7.
32. Inamoto N, Katsuya T, Kokubo Y, Mannami T, Asai T et al. (2003) Association of methylenetetrahydrofolate reductase gene polymorphism with carotid atherosclerosis depending on smoking status in a Japanese general population. *Stroke* 34(7):1628-33.
33. Devlin AM, Clarke R, Birks J, Evans JG, Halsted CH (2006) Interactions among polymorphisms in folate-metabolizing genes and serum total homocysteine concentrations in a healthy elderly population. *Am J Clin Nutr* 83(3):708-13.
34. Saw SM, Yuan JM, Ong CN. (2001) Genetic, dietary, and other lifestyle determinants of plasma homocysteine concentrations in middle-aged and older Chinese men and women in Singapore. *Am J Clin Nutr* 73(2):232-9.
35. Schwartz SM, Siscovick DS, Malinow MR, Rosendaal FR, Beverly RK et al. (1997) Myocardial infarction in young women in relation to plasma total homocysteine, folate, and a common variant in the methylenetetrahydrofolate reductase gene. *Circulation* 96(2):412-7.
36. Christensen B, Frosst P, Lussier-Cacan S et al. (1997) Correlation of a common mutation in the methylenetetrahydrofolate reductase gene with plasma homocysteine in patients with premature coronary artery disease. *Arterioscler Thromb Vasc Biol* 17(3):569-73.
37. Hustad S, Ueland PM, Vollset SE, Zhang Y, Bjorke-Monsen AL, Schneede J. (2000) Riboflavin as a determinant of plasma total homocysteine: effect modification by the methylenetetrahydrofolate reductase C677T polymorphism. *Clin Chem* 46(8 Pt 1):1065-71.
38. Meleady R, Ueland PM, Blom H, Whitehead SA, Refsum H et al. (2003) Thermolabile methylenetetrahydrofolate reductase, homocysteine, and cardiovascular disease risk: the European Concerted Action Project. *Am J Clin Nutr* 77(1):63-70.
39. Frederiksen J, Juul K, Grande P, Jensen GB, Schroeder TV et al. (2004) Methylenetetrahydrofolate reductase polymorphism (C677T), hyperhomocysteinemia, and risk of ischemic cardiovascular disease and venous thromboembolism: prospective and case-control studies from the Copenhagen City Heart Study. *Blood* 104(10):3046-51.
40. Nakai K, Fusazaki T, Suzuki T, Shiroto T, Osawa M et al. (2000) Genetic polymorphism of 5,10-methylenetetrahydrofolate increases risk of myocardial infarction and is correlated to elevated levels of homocysteine in the Japanese general population. *Coron Artery Dis* 11(1):47-51.
41. Kluijtmans LAJ, den Heijer M, Reitsma PH, Heil SG, Blom HJ et al. (1998) Thermolabile methylenetetrahydrofolate reductase and factor V Leiden in the risk of deep-vein thrombosis. *Thromb Haemost* 79(2):254-258
42. Tanis BC, Blom HJ, Bloemenkamp DG, van den Bosch MAAJ, Algra A et al. (2004) Folate, homocysteine levels, methylenetetrahydrofolate reductase (MTHFR) 677C --> T variant, and the risk of myocardial infarction in young women: effect of female hormones on homocysteine levels. *J Thromb Haemost* 2(1):35-41.
43. Passaro A, Vanini A, Calzoni F, Alberti L, Zamboni PF et al. (2001) Plasma homocysteine, methylenetetrahydrofolate reductase mutation and carotid damage in elderly healthy women. *Atherosclerosis* 157(1):175-80.
44. Bathum L, Petersen I, Christiansen L, Konieczna A, Sorensen TI et al. (2007) Genetic and environmental influences on plasma homocysteine: results from a Danish twin study. *Clin Chem* 53(5):971-9.
45. Jee SH, Song KS, Shim WH, Kim HK, Suh I et al. (2002) Major gene evidence after MTHFR-segregation analysis of serum homocysteine in families of patients undergoing coronary arteriography. *Hum Genet* 111(2):128-35.
46. Pullin CH, Ashfield-Watt PA, Burr ML, Clark ZE, Lewis MJ et al. (2001) Optimization of dietary folate or low-dose folic acid supplements lower homocysteine but do not enhance endothelial function in healthy adults, irrespective of the methylenetetrahydrofolate reductase (C677T) genotype. *J Am Coll Cardiol* 38(7):1799-805.
47. Litynski P, Loehrer F, Linder L, Todesco L, Fowler B (2002) Effect of low doses of 5-methyltetrahydrofolate and folic acid on plasma homocysteine in healthy subjects with or without the 677C-->T polymorphism of methylenetetrahydrofolate reductase. *Eur J Clin Invest* 32(9):662-8.
48. Ou T, Yamakawa-Kobayashi K, Arinami T, Amemiya H, Fujimara H et al. (1998) Methylenetetrahydrofolate reductase and apolipoprotein E polymorphisms are independent risk factors for coronary heart disease in Japanese: a case-control study. *Atherosclerosis* 137(1):23-8.
49. Jang Y, Park HY, Lee JH, Ryu HJ, Kim JY et al. (2002) A polymorphism of the methylenetetrahydrofolate reductase and methionine synthase gene in CAD patients: association with plasma folate, vitamin B12 and homocysteine. *Nutrition Research* 22(9):965-76.
50. Madonna P, de Stefano V, Coppola A, Cirillo F, Cerbone AM et al. (2002) Hyperhomocysteinemia and other inherited prothrombotic conditions in young adults with a history of ischemic stroke. *Stroke* 33(1):51-6.
51. Verhoef P, Kok FJ, Kluijtmans LA, Blom HJ, Refsum H et al. (1997) The 677C-->T mutation in the methylenetetrahydrofolate reductase gene: associations with plasma total homocysteine levels and risk of coronary atherosclerotic disease. *Atherosclerosis* 132(1):105-13.
52. D'Angelo A, Coppola A, Madonna P, Fermo I, Pagano A et al. (2000) The role of vitamin B12 in fasting hyperhomocysteinemia and its interaction with the homozygous C677T mutation of the methylenetetrahydrofolate reductase (MTHFR) gene. A case-control study of patients with early-onset thrombotic events. *Thromb Haemost* 83(4):563-70.
53. Zittoun J, Tonetti C, Bories D, Pignon JM, Tulliez M (1998) Plasma homocysteine levels related to interactions between folate status and methylenetetrahydrofolate reductase: a study in 52 healthy subjects. *Metabolism* 47(11):1413-8.
