# Supplementary material for: Reconciling the Evidence on Serum Homocysteine and Ischaemic Heart Disease: A Meta-Analysis
Source: PLoS One. 2011 Feb 2;6(2):e16473. doi: 10.1371/journal.pone.0016473 (PMC3032783; doi:10.1371/journal.pone.0016473)
Supplement: Table S4 — Citations of published randomised trials of B vitamins and ischaemic heart disease events included in the Meta-analysis ( Figure 3 ). (DOC) [file pone.0016473.s006.doc]

**Table S4:** Citations of published randomised trials of B vitamins and ischaemic heart disease events included in the Meta-analysis (Figure 3).

1. Baker F, Picton D, Blackwood S, Hunt J. (2002) Blinded comparison of folic acid and placebo in patients with ischemic heart disease: an outcome trial. *Circulation* 5 November vol 106(19) supplement
2. Albert CM, Cook NR, Gaziano M, Zaharris E, MacFadyen J et al. (2008) Effect of folic acid and B vitamins on risk of cardiovascular events and total mortality among women a high risk for cardiovascular disease: a randomized trial. *JAMA* 299(17):2027-2036
3. Toole JF, Malinow MR, Chambless LE, Spence JD, Pettigrew LC et al. (2004) Lowering homocysteine in patients with ischemic stroke to prevent recurrent stroke, myocardiao infarction, and death: The Vitamin Intervention for Stroke Prevention (VISP) Randomized Controlled Trial. *JAMA* 291:565-575
4. Zoungas S, McGrath BP, Branley P, Kerr PG, Muske C et al. (2006) Cardiovascular morbidity and mortality in the Atherosclerosis and Folic Acid Supplmentation Trial (ASFAST) in chronic renal failure. *J Am Coll Cardiol* 47(6):1108-1116
5. Liem A, Reynierse-Buitenwerf GH, Zwinderman AH, Jukema JW, van Veldhuisen DJ. (2003) Secondary prevention with folic acid: effects on clinical outcomes. *J Am Coll Cardiol* 41(12):2105-2113
6. Scnyder G, Roffi M, Flammer Y, Pin R, Hess OM et al. (2002) Effect of homocysteine-lowering therapy with folic acid, vitamin B_12_, and vitamin B_6_ on clinical outcome after percutaneous coronary intervention. The Swiss Heart Study: A Randomized Controlled Trial. *JAMA* 288:973-979
7. Ebbing M, Bleie O, Ueland PM, Nordrehaug JE, Nilsen DWet al. (2008) Mortality and cardiovascular events in patients treated with homocysteine-lowering B vitamins after coronary angiography: a randomized controlled trial. *JAMA* 300(7):795-804
8. The Heart Outcomes Prevention Evaluatiohn (HOPE) 2 Investigators (2006) Homocysteine lowering with folic acid and B vitamins in cardiovascular disease. *N Engl J Med* 354:1567-1577
9. Bonna KH, Njolstad I, Ueland PM, Schirmer H, Tverdal A et al. (2006) Homocysteine lowering and cardiovascular events after acute myocardial infarction. *N Engl J Med* 354:1578-1588
10. Study of the Effectiveness of Additional Reductions in Cholesterol and Homocysteine (SEARCH) Collaborative Group (2010) Effects of Homocysteine-Lowering With Folic Acid Plus Vitamin B12 vs Placebo on Mortality and Major Morbidity in Myocardial Infarction Survivors: A Randomized Trial, *JAMA* 303(24):2486-2494
11. Lange H, Suryapranata H, De Luca G, Börner C, Dille J et al. (2004) Folate therapy and in-stent restenosis after coronary stenting. N Engl J Med 350:2673-2681
12. House AA, Eliasziw M, Cattran DC, Oliver MJ, Dresser GK et al. (2010) Effect of B-vitamin therapy on progression of diabetic nephropathy: a randomized controlled trial. *JAMA* 303(16):1603-1609
13. Jamison RL, Hartigan P, Kaufman JS, Goldfarb DS, Warren SR et al. (2007) Effect of homocysteine lowering on mortality and vascular disease in advanced chronic kidney disease and end-stage renal disease: a randomized controlled trial *JAMA* 298(10):1163-1170
14. Righetti M, Serbelloni P, Milani S, Ferrario G (2006) Homocysteine-lowering vitamin B treatment decreases cardiovascular events in hemodialysis patients. *Blood Purif* 24:379-386
15. Study of the Effectiveness of Additional Reductions in Cholesterol and Homocysteine (SEARCH) Collaborative Group (2007) Characteristics of a randomized trial among 12064 myocardial infarction survivors. *Am Heart J* 154:815-823
